# Math Anxiety Is Related to Math Difficulties and Composed of Emotion Regulation and Anxiety Predisposition: A Network Analysis Study

**DOI:** 10.3390/brainsci11121609

**Published:** 2021-12-05

**Authors:** Lital Daches Cohen, Nachshon Korem, Orly Rubinsten

**Affiliations:** 1 Edmond J. Safra Brain Research Center for the Study of Learning Disabilities, Haifa 3498838, Israel; litaldaches@gmail.com (L.D.C.); nachshon.korem@yale.edu (N.K.); 2 Department of Learning Disabilities, University of Haifa, Haifa 3498838, Israel; 3 Department of Psychiatry, Yale University School of Medicine, New Haven, CT 06510, USA

**Keywords:** math anxiety, emotion regulation, reappraisal, suppression, math performance

## Abstract

Current evidence suggests emotion regulation is an important factor in both math anxiety and math performance, but the interplay between these constructs is unexamined. Given the multicomponent structure of math anxiety, emotion regulation, and math performance, here, we aimed to provide a comprehensive model of the underlying nature of the links between these latent variables. Using the innovative network analysis approach, the study visualized the underlying links between directly observable and measurable variables that might be masked by traditional statistical approaches. One hundred and seventeen adults completed a battery of tests and questionnaires on math anxiety, emotion regulation, and math performance. The results revealed: (1) state math anxiety (the emotional experience in math-related situations), rather than trait math anxiety, was linked to anxiety predisposition, subjective valence of math information, and difficulties in emotion regulation; (2) the link between state math anxiety and math performance partialed out the link between trait math anxiety and performance. The study innovatively demonstrates the need to differentiate between traits and tendencies to the actual emotional experience and emotion regulation used in math anxiety. The results have important implications for the theoretical understanding of math anxiety and future discussions and work in the field.

## 1. Introduction

Math anxiety is a common phenomenon [1] characterized by negative attitudes toward math [2,3], as well as feelings of stress, frustration, and fear when thinking about or engaging in situations involving numerical information [3,4,5]. Researchers have reported inconsistent results for the direction of the link between math anxiety and performance, e.g., [6,7,8]. A possible explanation often cited by intervention studies suggests that emotion regulation can explain the ability of some math-anxious individuals to perform well, e.g., [9,10,11]; however, see [12]. An additional factor that can explain the inconsistent results for the link between math anxiety and performance is that most studies have assessed a single math-anxiety variable, when, in fact, there are multidimensional interrelationships between many heterogeneous components [13], such as state and trait disposition [14].

Against this background, we wished to provide a comprehensive model of the underlying nature of the links between math anxiety, emotion regulation, and math performance. Given the multicomponent structure of math anxiety (i.e., state and trait math anxiety), emotion regulation (i.e., daily and spontaneous use of emotion-regulation strategies in math contexts), and math performance (i.e., distinct forms of math, such as calculation and fluency) [15], the innovative network analysis is a highly appropriate approach. Network analysis, a new framework for analyzing complex and abstract constructs, has recently gained traction in the field of mental disorders [16]. Our aims were twofold: (1) to reveal the relative and unique contribution of each measurable score/test to the complex network of interacting components that might be masked by traditional statistical approaches or interpreted in terms of broad, unobserved latent variables [17], and (2) to visualize the dynamics among constituent elements in the network [18].

### 1.1. State and Trait Math Anxiety

Although related to other anxiety subtypes, including general [19,20] and test anxiety [21,22], math anxiety is a unique anxiety disorder with a specific profile [21,23,24] and a distinct pattern of neural activity, e.g., [25]. Math anxiety was found to be linked to avoidance of math-related careers [3,26,27,28], increased health costs [29], reduced financial literacy [30], and low socioeconomic status [31]. The causes of math anxiety may be both intrinsic and environmental [32]. Intrinsic factors include brain malfunctions [33], genetic roots [34], and a tendency toward anxiety in general [19,20,35]. The proposed environmental causes include adverse pedagogical and social math-related events with teachers [36] and parents [37].

In line with the State–Trait Anxiety model [38], trait math anxiety refers to an acquired and sustained individual tendency to perceive math-related situations as threatening, whereas state math anxiety is a temporary anxiety response to specific situations which involves increased arousal of the autonomic nervous system. Self-report questionnaires that typically assess anxiety levels in hypothetical/retrospective math-related situations are the primary method for evaluating trait math anxiety [39]. In contrast, state math-anxiety assessments include real-time self-reports on the actual affective experience (i.e., current anxiety) in specific situations [40]. An additional indicator of state math anxiety may be the degree of pleasant and unpleasant feelings triggered by a math task (i.e., subjective valence) [41].

Although more vulnerable, people with trait anxiety do not necessarily experience state anxiety [42,43]. Accordingly, subjective self-report questionnaires do not always converge with objective unconscious cognitive constructs of math anxiety [37]. Thus, our study included self-reports on anxiety states in hypothetical/retrospective math-related situations (i.e., trait math anxiety) and real-time reports on current anxiety in situations involving numerical information (i.e., state math anxiety).

### 1.2. Anxiety Predispositions in Math-Anxious Individuals

The literature suggests the predisposition to general anxiety disorder is a risk factor for developing math anxiety [19,20,35]. For example, math-anxious individuals demonstrated general-anxiety-related neuro-physiological responses [44] and maladaptive threat-related attentional bias [45,46]. Moreover, genetic studies have found that general anxiety explained 9% of the total variance in math anxiety [34,47], and general and math anxiety shared about 20% of their genetic variance [48]. In a similar vein, a longitudinal path model showed general anxiety disorder was a significant predictor of math performance [44]. Yet, the etiology of math anxiety and the role general anxiety is playing in math anxiety are still missing [49].

### 1.3. Math Anxiety and Performance

Math anxiety has consistently been shown to be negatively related to math performance [50,51] within and across countries in PISA tests [52,53], with a stronger link in females [22]. This relationship is stable even when controlling for cognitive abilities, e.g., [3], working memory capacity [54], processing speed [55], and general anxiety [56]. In addition, no significant relationships between math anxiety and domain-remote skills such as reading, were evident [3]. Recent evidence suggests that state (or state-like) math anxiety, e.g., [6,44,57,58,59] is more associated with math performance than trait math anxiety, e.g., [40,58,60,61,62,63], and the link between math anxiety and math performance is stronger in more complex math tests [64,65,66]. However, only a small number of studies used multiple measures of math performance [67].

Findings on the direction of causality between math anxiety and performance suggest a reciprocal link [57,68]. Math anxiety predicts decreased achievements [44,64], which, in turn, predict the development of math anxiety [44,69,70]. This reciprocal relationship between math anxiety and performance may be driven by an avoidance behavior which increases emotional [71,72] and learning problems [57,73,74]. However, this vicious cycle can be interrupted [6,7,8], a phenomenon that may be explained by emotion-regulation processes [7,10,33]. For example, increased activation in the fronto-parietal network associated with cognitive control and emotion regulation prior to a math task leads to higher achievement among highly math-anxious individuals [75]. Given the interesting findings on the role of emotion regulation in coping with math anxiety, we wished to further investigate the interplay between math anxiety, emotion regulation, and math performance while considering general anxiety.

### 1.4. Links between Emotion Regulation and Math Anxiety

Emotion regulation is a mental process that consciously or unconsciously [76,77] affects the type, duration, intensity, and expression of emotions [78,79] in order to accommodate environmental demands [79]. Two widely studied emotion-regulation strategies are cognitive reappraisal and expressive suppression [79,80]. *Cognitive reappraisal* constitutes an antecedent-focused strategy that aims to modify thoughts and beliefs about a stimulus or situation in a way that alters the emotional response to it [79]. A habitual use of reappraisal leads to a decrease in the subjective experience of negative emotions and an increase in adaptive responses to emotionally evocative events [79,81]. Expressive suppression, in contrast, is a response-focused strategy in which the individual attempts to conceal his/her feelings, behaviors, and physiological activity. People who tend to use suppression in daily life are reported to have decreased subjective positive effect [79]. Not surprisingly, then, suppression is consistently associated with anxiety disorders, whereas reappraisal has been linked to increased resilience to the development of anxiety disorders [82,83,84,85].

Previous studies have indicated that the ability to regulate emotions predicts the severity of anxiety-related symptoms up to five years [86] and also predicts subsequent achievements [87], suggesting it may play a central role in the relations between math anxiety and math performance. Indeed, math anxiety involves difficulties in emotion regulation [7,33,88,89,90]. For example, in one study, highly math-anxious individuals demonstrated decreased activation in areas necessary for solving math problems (i.e., the dorsolateral prefrontal cortex) and increased activation in competing networks related to emotional processing (i.e., the default mode network) [25].

By manipulating emotion regulation, previous studies showed that reappraisal can reduce math-anxiety reactions [8,91,92] and improve math performance [8,9,10,11,91,93]; however, see [12].People implement multiple regulatory strategies in any given emotional episode [94], a phenomenon termed emotion polyregulation [95]. 

In our study, we innovatively investigated the way trait (daily) and state (actual use of) emotion-regulation strategies during a math test and trait and state math anxiety interacted with each other simultaneously and affected performance in calculation accuracy and math fluency tests. 

### 1.5. The Current Study

The construct of math anxiety has received increasing attention in recent years [23] because of its close relations with math performance [3,57] and its far-reaching consequences, e.g., [3,27,30]. Emotion regulation, particularly reappraisal, has been shown to be effective in reducing math-anxiety reactions [8,91,92] and in improving math performance [8,9,10,11,91,93]; however, see [12]. To strengthen the effectiveness of interventions, it is necessary to ecologically describe the drivers of the link between the latent variables, and to assess the different contributions of trait and state measures of math anxiety, emotion regulation, general anxiety, and math performance to the network.

Through ecological descriptive research and the use of a powerful statistical approach, we investigated the correlations between state and trait math anxiety, state and trait emotion regulation, and performance in distinct forms of math, and analyzed the unique contribution of each observable measure to the larger network. We also assessed and visualized the dynamics among constituent elements of this complex network [18]. The findings complement those of classic models that collapsed these complex traits into abstract variables [96] and provide a comprehensive model of the underlying nature of the links between math anxiety, emotion regulation, general anxiety, and math performance.

In line with the reviewed literature, we hypothesized that: (1) math anxiety would be associated with difficulties in emotion regulation [7,8,25,33,88,89,90] and with state and trait general anxiety [35,44,48]; (2) the link between math anxiety and performance would be stronger for state, e.g., [6,44,58] rather than trait math anxiety, e.g., [40,58]; (3) math performance would be positively linked to reappraisal [8,9,10,11,91,93]; however, see [12].

## 2. Method

### 2.1. Participants

We calculated sample size according to the previously reported correlation between state math anxiety and math achievements [97]. Based on *r* = −0.27, power = 0.8, and a significance level of 0.05, we concluded 105 subjects would be sufficient for our analysis. Participants included 134 adults (95 females; M = 28.38 years, SD = 4.35). Participants had different seniority in university studies. All had normal or corrected-to-normal vision and no history of neurologically based impairments, such as ADHD, or learning disabilities (e.g., dyslexia and dyscalculia). Prior to data collection, participants signed a consent form approved by the University of Haifa ethics committee (429/17, date of approval: 11 June 2019). 

### 2.2. Measures

The questionnaires were translated by the author into Hebrew (forward translation) and from Hebrew back to English (back translation) to ensure the validity of the translations.

#### 2.2.1. Trait Math Anxiety 

We used the Hebrew-translated versions of the Abbreviated Math Anxiety Scale (AMAS) [98] to measure trait math anxiety. The AMAS is a nine-item self-report questionnaire found to be as effective as the longer Mathematics Anxiety Rating Scale [98]. Each item consists of a statement describing an event; participants indicate how anxious it would make them on a five-point Likert scale from *never* to *always*. Scores on the AMAS range from 9 to 45, with a higher score indicating a higher level of math anxiety. Cronbach’s alpha for the AMAS in our sample was 0.92.

#### 2.2.2. State Math Anxiety 

State anxiety during a math test was assessed by the state-Mathematics Anxiety Questionnaire (state-MAQ) [97]. The questionnaire was developed from the State Anxiety Inventory [99]. Using a four-point Likert scale ranging from *not at all* to *very much*, participants indicate whether an emotional state applies to them immediately before a math test (pre-test; seven items) or has done so during a math test (post-test; seven items). The total score was obtained by summing each item rating. To control other influential factors, participants were instructed to fill out the questionnaire only in the light of the upcoming/completed math test and not consider other circumstances. The reliability was α = 0.92.

#### 2.2.3. Subjective Valence Ratings of Math Information 

Similar to previous research [100], we asked participants to rate how they felt on a visual analogue scale (VAS) ranging from *very bad* to *very good* using the mouse immediately after a math test. While response bias is inevitable in scientific research, we used the reliable VAS [101] to capture small yet persistent effects in subjective emotional states [101,102,103,104].

#### 2.2.4. State/Trait Anxiety 

The Spielberger’s State–Trait Anxiety Inventory (STAI) [99] was used to measure and differentiate between anxiety as a trait and a state. The STAI state scale consists of 20 statements asking people to describe how they feel at a particular moment in time (e.g., calm, tense) rated on a four-point intensity scale from *not at all* to *very much*. The STAI trait scale consists of 20 statements describing how people generally feel (e.g., confident), rated on a four-point frequency scale from *almost never* to *almost always*. The total score of each subscale was obtained by summing each item rating. Internal consistencies for the state scale scores ranged from 0.83 to 0.92 for male and female high-school and college students; for the trait-scale scores, coefficients of internal consistency ranged from 0.86 to 0.92 [99]. In our sample, the coefficient alpha for the state and the trait scales were 0.92 and 0.91, respectively. 

#### 2.2.5. Daily Use of Reappraisal and Suppression 

The well-known Emotion Regulation Questionnaire (ERQ) [105] was used to measure the frequency of the habitual use of reappraisal and suppression. The ERQ has acceptable validity and reliability [105]. It includes 10 items, six of which measure reappraisal frequency (e.g., “I control my emotions by changing the way I think about the situation I’m in”) and four expressive suppression frequency (e.g., “I control my emotions by not expressing them”). Items are rated on a seven-point Likert-type response scale from *strongly disagree* to *strongly agree*. Higher scores on an item indicate greater use of the corresponding strategy. We used the average score of the relevant subscale to create the total score of the frequency of the use of each strategy (appraisal, suppression). The coefficient alpha for the ERQ in our sample was 0.83.

#### 2.2.6. Spontaneous Use of Reappraisal and Suppression

We administered a six-item scale based on the ERQ [105] to measure spontaneous use of reappraisal (three items) and suppression (three items) [106], with responses ranging from *not at all* to *very much*. Participants indicated which emotion-regulation behavior they used during a math task (post-test). The coefficient alpha for the scale in our sample was 0.70.

#### 2.2.7. Difficulties in Emotion Regulation

The Difficulties in Emotion Regulation scale (DERS) [107] is a 36-item scale developed to assess multiple facets of emotion regulation, on a five-point Likert scale ranging from *almost never* to *almost always*. Higher scores indicate greater difficulties in emotion regulation. The DERS has high internal consistency, good test–retest reliability, and adequate construct and predictive validity [107]. The coefficient alpha for the DERS in our sample was 0.92.

#### 2.2.8. Math Performance

We used the *Math Fluency* and *Calculation* subtests of the Woodcock–Johnson III Tests of Achievement [108] to measure math performance. *Math Fluency* assesses automaticity with basic arithmetic facts. In this subtest, participants are required to quickly and accurately complete simple arithmetic problems within a three-minute time limit. Arithmetic computations include simple addition, subtraction, and multiplication operations. The *Calculation* subtest measures the ability to perform increasingly difficult math computations, from simple addition to advanced geometry and trigonometry, with no time limit. The number of correctly completed computations was totaled in each subtest.

### 2.3. Procedure

Due to COVID-19 restrictions in Israel, most participants (~70%) were recruited through iPanel (iPanel.co.il; iPanel, Bnei-Brak, Israel), an online Israeli pooling service, from June to September 2021, and a small proportion of participants (~30%) were recruited through invitations posted on various student internet groups and forums [109,110,111]. iPanel can deliver a representative sample of the adult Jewish population of Israel while adhering to the stringent standards of the European Society for Opinion and Marketing Research (ESOMAR). In addition, iPanel was evaluated by the Applied Statistical Laboratory of the Hebrew University of Jerusalem and found to be highly accurate [112]. Participants who registered in iPanel who met the inclusion criteria and participants recruited through the Internet received an invitation to participate and gave their consent. Participants who registered in iPanel received vouchers from the survey company in exchange for their participation. Participants recruited through the Internet received monetary compensation of a sum equivalent to USD 10.

The study was administered via E-Prime Go Software. First, the AMAS questionnaires were randomly presented, followed by the state-MAQ (pre-test) and the fluency and calculation subtests in random order. In the fluency and calculation subtests, arithmetic problems and math computations appeared one after the other when the participant pressed the space button to move on to the next one. *A* previous study found no significant differences between an online, remote administration procedure and traditional, in-person administration of the Woodcock–Johnson IV cognitive ability and academic achievement tests [112]. Then, the state-MAQ (post-test) and spontaneous use of reappraisal and suppression questionnaires were randomly administered. Next, the STAI, ERQ, and DERS questionnaires were presented in random order. The state-MAQ addressed an emotional state before (pre-test) or during (post-test) the math tests, whereas the STAI referred to general emotional states. Throughout the study, general questions (e.g., gender, age, time) were presented in order to ensure the validity of responses. Note that this was part of an ongoing larger study including additional cognitive tasks and emotional questionnaires.

### 2.4. Network Analysis

The entire network analysis procedure was based on Epskamp and Fried [113]. We used the R-package qgraph to estimate and visualize the network [114]. We estimated Gaussian graphical models (GGMs). In these models, each test is represented by a node in the network, and the edges represent partial correlation coefficients between the nodes using a color code (red for negative and blue for positive relations), with edge thickness representing the strength of a direct interaction between two nodes [115]. Based on Epskamp and Fried’s [113] guidelines, we computed the network using the least absolute shrinkage and selection operator (LASSO). LASSO is used to reduce false-positive edges and to deal with problems such as multicollinearity [116]. It identifies the edges that differ significantly from zero and most accurately reveal the underlying network (i.e., every edge that survives the GLASSO regression is significant). The tuning parameter (gamma) for the GLASSO estimation in our experiment was 0.5. A tuning parameter is chosen to minimize the extended Bayesian Information Criterion parameter and has been shown to accurately recover underlying network structures [117]. We determined node placement using the Fruchterman and Reingold’s [118] algorithm, which places connected nodes closer to each other and more connected nodes closer to the center.

## 3. Results

The final sample consisted of 117 adults (85 females; M = 28.38 years, SD = 4.35). A total of 7 males and 10 females were removed from analysis because their RTs or accuracy rates were outliers (below or above 1 SD) and/or due to missing values. Descriptive statistics of research variables are presented in Table 1. The correlation matrix is presented in Table 2. The results replicate previous findings by showing: (1) positive correlations between math and general anxiety [19,20]; (2) positive correlations between state- and trait-anxiety measures [42,43]; (3) negative correlations between math anxiety and math performance, e.g., [3,57]; (4) positive correlations between reappraisal and anxiety measures, e.g., [8,92]; (5) negative correlations between reappraisal and math performance, e.g., [8,10]. As the correlation matrix in Table 2 shows, anxiety and performance “in cluster” correlations were significant (i.e., within traditional latent constructs).

**Figure 1:**
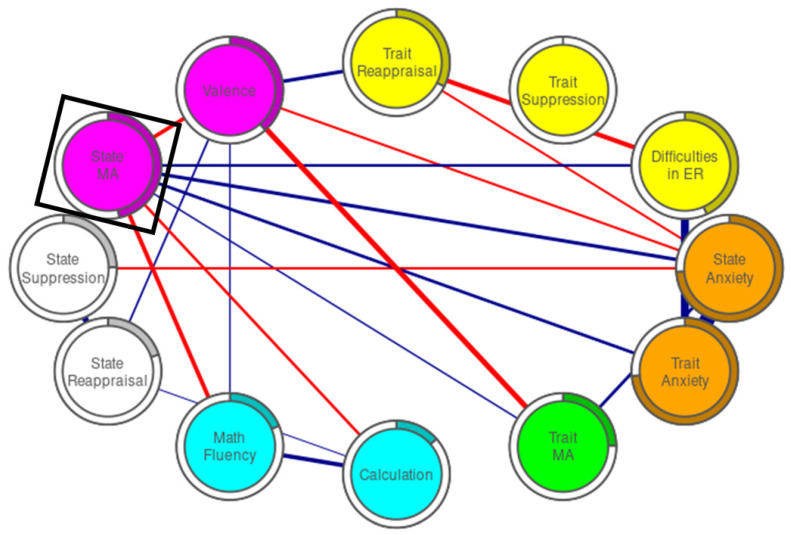
Regularized partial correlations network. Edge thickness represents the strength of a direct interaction between two nodes. Blue and red edges indicate positive and negative relations, respectively. The black square represents the focus of the study. The orange nodes represent the general-anxiety subscales (the STAI subscales). The green node represents the trait math-anxiety questionnaire (AMAS questionnaire). The magenta nodes represent state math-anxiety measures (State MA = state-MAQ questionnaire, Valence = valence ratings of math information). Cyan nodes represent math tests. The yellow nodes represent daily use of emotion-regulation strategies (the ERQ subscales) and difficulties in emotion regulation (DERS questionnaire). White nodes represent spontaneous use of reappraisal and suppression strategies during math tests. The painted area in the rings around the nodes depicts predictability.

The GGM network is visualized in Figure 1. The network is composed of the following five theoretically assumed clusters: general anxiety (i.e., state and trait anxiety), state math anxiety and valence rating, math performance (i.e., math fluency and calculation), trait emotion regulation (i.e., daily and spontaneous use of and difficulties in emotion regulation; note that trait suppression was partial out of the network), and state emotion regulation. Of the possible 78 edges (i.e., links), only 21 were retained (for 95% CI around the edge, see Supplementary Figure S1). Predictability (i.e., variance of a node explained by its neighbors) ranged from 0% in the trait-suppression scale (i.e., daily use of suppression measure) to 74.3% in the state anxiety, and average predictability was 34%. 

Four results are noteworthy. First, state math anxiety edges to performance partialed out the trait math-anxiety edges. Second, state math anxiety was positively linked to both state and trait anxiety. Third, spontaneous use of reappraisal during math tests was positively linked to subjective valence ratings of math information and calculations, whereas trait reappraisal had no direct links to state or trait math anxiety or math performance. Fourth, both state and trait math-anxiety questionnaires were linked to valence ratings of math information.

To sum up, our results clearly show that state math anxiety (i.e., a temporary and math-situation-related anxiety reaction) consists of predisposition to anxiety in general, negative subjective valence of math information, difficulties in emotion regulation, and is associated with low math performance.

## 4. Discussion

The primary purpose was to provide a comprehensive model of the nature of the links between math anxiety, emotion regulation, general anxiety, and math performance. Our powerful statistical approach enabled us to distinguish between the unique contribution of each observable variable [96] and visualize the dynamics among constituent elements in the network [18]. Here, we show that the emotional experience during math-related situations is tightly linked to the tendency toward anxiety in general, the degree of pleasant and unpleasant feelings triggered by a math task (i.e., subjective valence) [41], and difficulties in emotion regulation.

Importantly, the link between state math anxiety and math performance partialed out the link between trait math anxiety and performance. These results are consistent with previous findings of stable negative correlations between state (or state-like) math anxiety and math performance, e.g., [6,44,57,58,59]. They are also consistent with findings of a lack of correlations between fear of failure in math (i.e., trait math anxiety) and performance, e.g., [40,58,60,61,62,63]. However, math anxiety is commonly described as a trait [39]. Although related, state math anxiety, which refers to a temporary and math situation-related anxiety reaction, has been found to be less subject to bias [119] and more related to attitudes and motivation [58]. Note that in contrast to a small number of studies [64,65,66], we did not find differences in the math anxiety–performance link with various aspects of math (calculation and fluency). More research is needed to comprehensively test these relations [67].

Innovatively, we showed the spontaneous use of reappraisal in math-related situations, but not the tendency to use reappraisal in daily life, was positively linked to subjective valence ratings of math information and math performance. These findings are consistent with evidence of difficulties in emotion regulation in math anxiety [7,25,33,88,89,90] and explain the success of focused and brief reappraisal interventions in reducing math-anxiety reactions [8,91,92] and improving math performance [8,9,10,11,91,93]; however, see [12]. They are also consistent with the emotion-polyregulation phenomenon [95], according to which people implement various and multiple regulatory strategies in a given emotional episode [94].

Taken together, the study highlights for the first time the need to differentiate between general traits (i.e., general anxiety and trait math anxiety) and tendencies (i.e., daily use of emotion-regulation strategies), and the actual emotional experience (i.e., state math anxiety and subjective valence ratings) and emotion-regulation use in specific math contexts (i.e., spontaneous use of emotion-regulation strategies during a math test). From a practical perspective, the combined examination of general-anxiety predispositions, the subjective valence of math information, and difficulties in emotion regulation can help psychologists and educators optimally tailor an intervention plan to a specific case and maximize its outcomes. Importantly, the current study highlights the central role of emotion regulation in educational processes and suggests providing educators practical tools ways to promote the use of adaptive emotion-regulation strategy (i.e., reappraisal) in pedagogical programs. Finally, the study indicates the need to focus on promoting the use of reappraisal in specific math-related situations to reduce anxiety levels and improve achievement among people who experience a heightened negative emotional reaction in these contexts.

## 5. Limitations

The study makes an important first step towards an understanding of the interplay between math anxiety, emotion regulation, and math performance and highlights the need to distinguish between general traits and tendencies and the actual emotional experience in math-related situations. However, there are several limitations. First, recruiting participants via the Internet and social networks makes it difficult to further generalize the results and may threaten the findings’ reliability and validity, e.g., [120]. In many studies, however, internet-based data were shown to have high reliability, replicability, and theoretical consistency comparable to data gathered in a traditional lab setting, e.g., [121,122]. In this study, the coefficient alphas for the self-report questionnaires ranged from 0.70 in the spontaneous use of reappraisal and suppression questionnaire to 0.92 in the AMAS, state-MAQ, state-anxiety scale, and DERS.

Second, only adults with different seniority in university studies participated, with twice as many females as males. Future studies will have to test the model in different age groups, as well as in the general (non-student) population, with more participants and a balanced number of females and males. A recent meta-analysis [51] suggests the math anxiety–performance link is stronger among senior high-school students, followed by junior high-school, university, and elementary students. These differences might influence the edges between math anxiety, emotion regulation, and math performance.

Third, the network approach lacks directionality even though it expands our understanding of the origins of the links between latent variables. Future research should address causal inferences and consider assessing state math anxiety through implicit measures, such as Skin Conductance Response and Heart Rate Variability, e.g., [123], to assess inaccessible cognitive and emotional structures that are processed automatically [124].

## 6. Conclusions

The research visualizes the interplay between math anxiety, emotion regulation, and math performance. As we opted to use partial correlations between observable tests instead of latent models, our findings demonstrate, first, that the temporary and math-situation-related anxiety reaction consists of a predisposition to general anxiety, negative subjective valence of math information, and difficulties in emotion regulation. Second, the state math anxiety edge to math performance partialed out the edge between trait math anxiety and performance. Third, instead of focusing on general traits (i.e., general anxiety and trait math anxiety) and tendencies (i.e., daily use of emotion regulation), more emphasis should be put on the actual emotional experience (i.e., state math anxiety and subjective valence ratings) and emotion-regulation use in specific math contexts (e.g., spontaneous use of emotion regulation during a math test). Future research should carefully consider the relationships between directly measurable variables of math anxiety and emotion regulation that might be masked by traditional statistical approaches or interpreted in terms of unobserved latent variables.

## Figures and Tables

**Table 1:** Descriptive statistics of research variables.

Latent Variable		Test	Mean (*SD*)	Min–Max
Emotion regulation	Daily use of emotion regulation	ERQ reappraisal scale	4.62 (1.27)	1–7
		ERQ suppression scale	3.41 (1.50)	1–7
	Difficulties in emotion regulation	DERS	86.37 (21.28)	40–149
	Spontaneous use of emotion regulation during math tests	Spontaneous reappraisal	3.26 (0.97)	0.33–5
		Spontaneous suppression	2.99 (1.02)	0.33–5
Anxiety	General anxiety	State anxiety	41.73 (11.91)	20–73
		Trait anxiety	40.65 (10.93)	20–76
	Trait math anxiety	AMAS	24.22 (8.49)	9–44
	State math anxiety	State-MAQ	2.31 (1.25)	0–5.71
		Valence ratings	13.27 (3.70)	2–18
Math performance		Fluency	100.30 (22.02)	54–159
		Calculation	20.39 (6.23)	8–40

**Table 2:** Correlation matrix of research variables.

Variable	1	2	3	4	5	6	7	8	9	10	11
1. ERQ reappraisal											
2. ERQ suppression	0.13										
3. DERS	−0.43 ***	0.09									
4. Spontaneous reappraisal	0.15	−0.02	−0.06								
5. Spontaneous suppression	0.14	−0.10	−0.06	0.37 ***							
6. State anxiety	−0.41 ***	−0.05	0.55 ***	−0.23 *	−0.29 **						
7. Trait anxiety	−0.39 ***	−0.01	0.63 ***	−0.19 *	−0.18	0.83 ***					
8. AMAS	−0.08	−0.10	0.14	−0.15	−0.16	0.42 ***	0.36 ***				
9. State-MAQ	−0.34 ***	−0.00	0.46 ***	−0.14	−0.01	0.56 ***	0.55 ***	0.36 ***			
10. Valence	0.37 ***	16	−0.26 **	0.24 **	0.08	−0.46 ***	−0.42 ***	−0.44 ***	−0.46 ***		
11. Fluency	0.02	−0.01	−0.11	0.13	0.41	−0.19 *	−0.17	−0.25 **	−0.38 ***	0.29 **	
12. Calculation	0.03	0.11	−0.06	0.19 *	−0.12	−0.09	−0.06	−0.22 *	−0.32 ***	0.26 **	0.34 ***

*Note*. * *p* < 0.05, ** *p* < 0.01, *** *p* < 0.001.

## Data Availability

The data and materials are available at https://osf.io/hj56d/ (accessed on 30 November 2021).

## Supplementary Materials

The following are available online at https://www.mdpi.com/article/10.3390/brainsci11121609/s1, Figure S1: Bootstrapped confidence intervals of the edge weights in the network.
