# Supplementary material for: Math Anxiety Is Related to Math Difficulties and Composed of Emotion Regulation and Anxiety Predisposition: A Network Analysis Study
Source: Brain Sci. 2021 Dec 5;11(12):1609. doi: 10.3390/brainsci11121609 (PMC8699086; doi:10.3390/brainsci11121609)

Supplementary Materials

Supplementary Figure S1. Bootstrapped confidence intervals of the edge weights in the network.

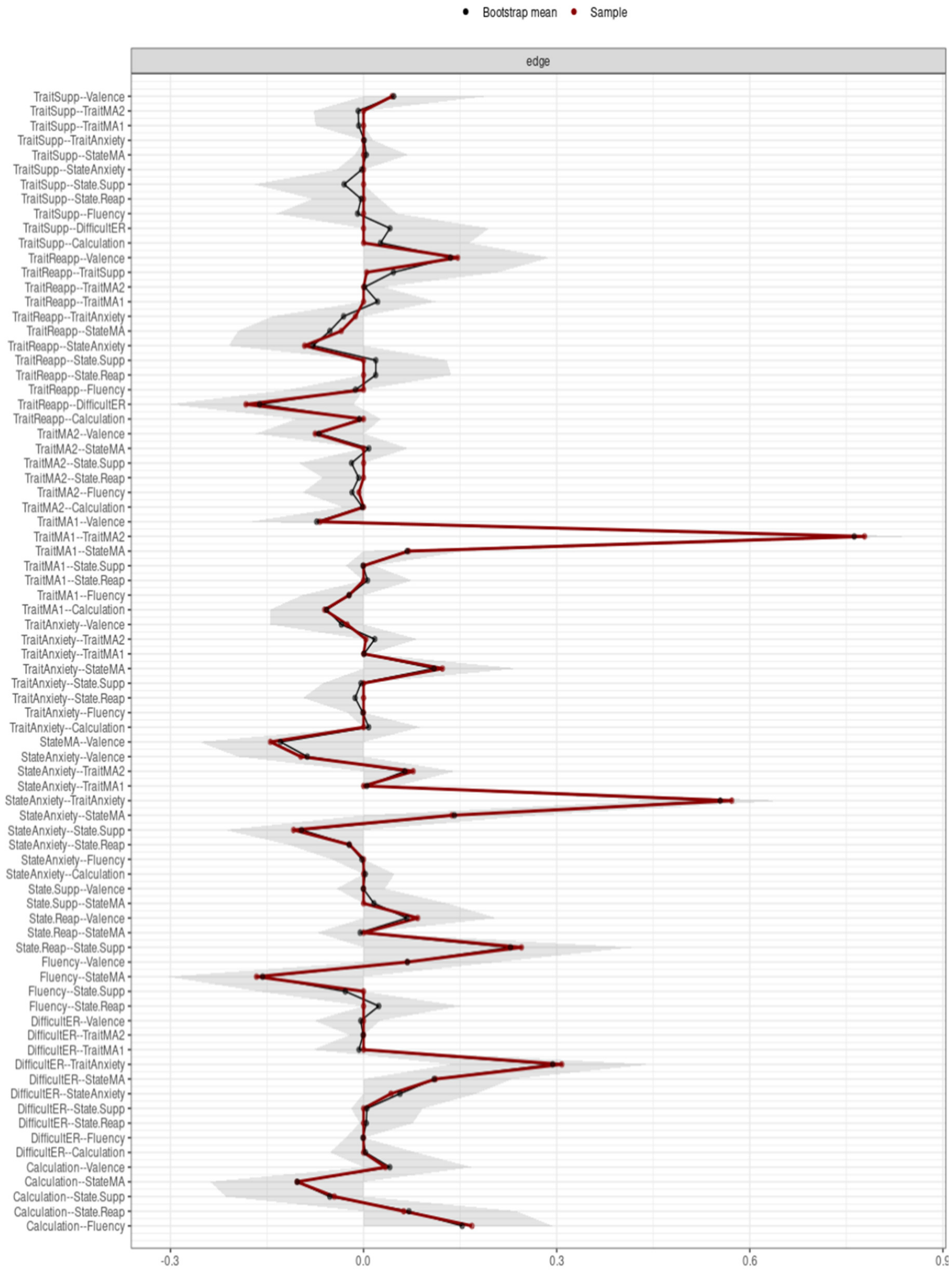

Supplement: Supplementary file 1 [file brainsci-11-01609-s001.zip › brainsci-1463172-supplementary.pdf]
